# Predictors of Survival in Atypical Meningiomas

**DOI:** 10.3390/cancers13081970

**Published:** 2021-04-21

**Authors:** Michele Da Broi, Paola Borrelli, Torstein R. Meling

**Affiliations:** 1 Faculty of Medicine, University of Oslo, 0372 Oslo, Norway; michele.dabroi@studenti.univr.it; 2 Department of Neurosurgery, Oslo University Hospital, 0372 Oslo, Norway; 3 Laboratory of Biostatistics, Department of Medical, Oral, and Biotechnological Sciences, University “G. d’Annunzio” Chieti-Pescara, 66100 Chieti, Italy; paola.borrelli@unich.it; 4 Department of Clinical Neurosciences, Division of Neurosurgery, Geneva University Hospitals, 1205 Geneva, Switzerland; 5 Faculty of Medicine, University of Geneva, 1206 Geneva, Switzerland

**Keywords:** intracranial tumor, atypical meningioma, neurosurgery, retreatment-free survival, recurrence rate, gross total resection

## Abstract

**Simple Summary:**

Meningiomas are the most common intracranial tumor [1] and are classified by the World Health Organization (WHO) as grade I (benign), grade II (atypical), or grade III (anaplastic) [2]. Regarding atypical meningiomas, predictors of overall survival (OS) and progression-free survival (PFS) are less well documented compared to their benign counterparts. Moreover, one of the most critical aspects of meningiomas is tumor relapse/progression that may also take place after the complete removal of the lesion. Recurrent lesions pose the question whether it is reasonable to perform second surgery. Alternative approaches include radiotherapy (RT) (stereotactic radiosurgery or conventional fractionated RT). We investigated 77 consecutive patients who underwent craniotomy for intracranial atypical meningiomas to evaluate predictors of OS and retreatment-free survival, and to assess the benefits of surgical retreatment for subsequent recurrences. We concluded that gross total resection (GTR) significantly prolonged retreatment-free survival but had no significant impact on OS. GTR was also associated with improved/stable neurological outcomes at 6–12 months. Age at surgery, preoperative Karnofsky performance scale (KPS), and retreatment were all strong prognostic factors of OS. Time-to-retreatment did not decrease significantly in patients requiring repeated surgical excision.

**Abstract:**

Introduction: Predictors of survival and progression of disease in atypical meningiomas are less well documented in the literature compared to benign meningiomas. Higher grade meningiomas tend to recur often and one of the most critical aspects is how to best deal with relapses. Methods: A total of 77 consecutive patients who underwent craniotomy for atypical meningioma between 1990–2010 at Oslo University Hospital (OUH) were reviewed. Results: Median age at surgery was 62.21 [interquartile range (IQR): 22.87] years. Fifty-one patients (66.2%) had neurological deficits at presentation. Fifty-four patients (70.1%) underwent gross total resection (GTR). Thirty-nine patients (50.7%) had improved/stable neurological outcomes at 6–12 months. Twenty-two patients (28.6%) underwent retreatment, of whom 20 (26.0%) were subjected to resection followed by adjuvant radiotherapy. Overall survival (OS) was significantly longer in patients <65 years (*p* < 0.001), with preoperative Karnofsky performance scale (KPS) score of ≥ 70 (*p* = 0.006), and who required no retreatment (*p* = 0.033). GTR significantly prolonged the retreatment-free survival rate (*p* < 0.001). STR carried almost a six-fold greater risk of neurological outcome deterioration (*p* = 0.044). Conclusions: GTR significantly prolonged retreatment-free survival but had no significant impact on OS. STR was a significant risk factor for deteriorated neurological outcome. Age, preoperative KPS, and retreatment were all strong predictors of OS. Median time-to-retreatment (TTR) did not shorten significantly throughout repeated surgeries.

## 1. Introduction

Meningiomas are the most common intracranial tumor originating from the arachnoid cap cells [1], and are classified by the World Health Organization (WHO) as grade I (benign), grade II (atypical), or grade III (anaplastic) [2]. Most meningiomas are benign and when operative management is required, surgical resection is the first-choice treatment, aiming at gross total resection (GTR) [3,4,5,6,7,8,9]. In case of asymptomatic meningiomas, however, an observational clinico-radiological follow-up may be preferable. Although the prognosis for patients with benign meningiomas is generally very favorable, outcomes for patients with high grade meningiomas are poorer [4]. With regard to atypical meningiomas, predictors of overall survival (OS) and progression-free survival (PFS) are less well documented compared to their benign counterparts. Several authors have identified GTR as a predictor of prolonged PFS [10,11], while its impact on OS is less clear [10,12,13,14]. Similarly, advanced age at surgery and bony involvement have been reported to be negative prognostic factors of OS [15,16,17].

One of the most critical aspects of meningioma surgery is tumor relapse/progression. As a matter of fact, recurrent lesions pose the question whether it is reasonable to undertake a second operation, or if it is more appropriate to use less invasive strategies, for instance conventional fractionated radiotherapy. This is particularly true when it comes to high grade meningiomas, since they are very prone to relapse. For atypical meningiomas, long-term recurrence rates identified by Aghi et al. [18] were 41% at 5-year and 48% at 10-year. According to Hammouche et al. [19], 1-year recurrence-free survival was 92% and fell to 53% at 5-year. According to a recent publication by Lemeè et al. [20], which included both low and high grade meningiomas, the benefit of the surgical treatment declines with the number of surgeries, since the time-to-retreatment (TTR) decreases significantly between surgeries in patients undergoing repeat resections.

Our objective was to use a retrospective cohort study design to analyze the clinical course of patients carrying atypical meningiomas treated at the Oslo University Hospital (OUH) to evaluate predictors of OS and retreatment-free survival, and to assess the benefits of surgical retreatment for subsequent recurrences.

## 2. Results

### 2.1 Overall Characteristics

The present study included 77 consecutive patients who underwent craniotomy for intracranial atypical meningiomas (WHO grade II). Median follow-up was 5.75 [interquartile range (IQR): 8.14] years and no patient was lost to follow-up. The female-to-male ratio was 1.5 and the median age at primary surgery was 62.21 [IQR: 22.87] years. Median Karnofsky Performance Score (KPS) was 80 [IQR: 20]. Fifty-one patients (66.2%) had neurological deficits at presentation, 36 cases (46.8%) showed symptoms related to increased intracranial pressure (ICP), 21 patients (27.3%) presented with preoperative seizures, and only 1 patient (1.3%) was asymptomatic. Twenty-four cases (31.2%) had skull base meningioma (SBM) according to Al-Mefty’s definition [21]. Bone invasion was detected in 17 meningiomas (22.1%) and 5 patients (6.5%) had multiple meningiomas according to preoperative imaging (Table 1).

### 2.2 Surgical and Neurological Outcomes

All patients in the present cohort underwent surgery aiming at the achievement of GTR. Simpson grade I and II resections were attained in 35 (45.5%) and 19 patients (24.7%), respectively. Altogether, 23 cases (29.9%) received partial resection. Of those, only 1 (1.3%) underwent adjuvant radiation therapy (RT). No patient died within 30 days of surgery. Two patients (2.6%) had early postoperative hematoma that required surgical evacuation and 4 (5.2%) had postoperative infection. Neurological status at 6–12 months improved or remained stable in 39 individuals (50.6%), while it worsened compared to preoperative status in 9 patients (11.7%) (Table 2).

Overall, 22 patients (28.6%) underwent retreatment after primary surgery by means of surgery alone in 1 case (4.6%), RT alone in 1 case (4.6%), and surgery followed by RT in 20 cases (90.9%). After these procedures, only 1 patient (4.5%) presented an infection that needed surgical management. No other complications were recorded (Table 2). Histological analysis after second surgery documented the transformation of 5 atypical meningiomas (22.7%) into anaplastic tumors (WHO grade III) (Table 2).

Thereafter, 11 patients (14.3%) underwent a third surgical resection for symptomatic meningioma relapse. Only 1 postoperative infection (9.1%) was recorded and pathological investigation showed another transformation to WHO grade III lesion. Four patients (5.2%) underwent a fourth surgery and two patients (2.6%) underwent a total of six tumor excisions during the timeframe of the study.

The median TTR from primary surgery to retreatment was 1.61 [IQR: 3.85] years, while the median TTR from retreatment to second retreatment was 1.62 [IQR: 2.13] years and the difference was not statistically significant (*p* = 0.836).

### 2.3 Predictors of Overall Survival and Retreatment-Free Survival

**Figure 1:**
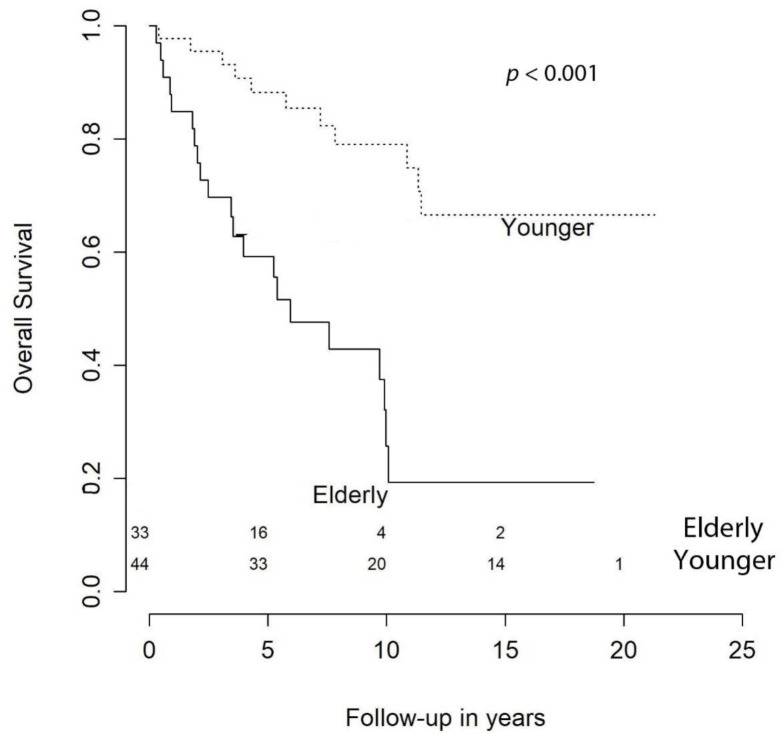
Overall survival by age.

With respect to OS, at 1 year it was 94.5%, at 2 years it was 91.8%, at 3 syear it was 86.3%, at 5 years it was 81.9%, at 10 years it was 65.7%, and at 15 years it was 56.6%. Our univariate analysis showed that age at primary surgery (HR (hazard ratio) = 1.07 [CI (confidence interval) 95%, 1.03–1.10], *p* < 0.001), preoperative KPS < 70 (HR = 3.70 [CI 95%, 1.51–9.09], *p* = 0.005), meningiomas requiring retreatment (HR = 2.13 [CI 95%, 1.06–4.28], *p* = 0.033), and subtotal resection (STR) were negative predictors of OS (Figure 1). The predictive power of age at surgery, preoperative KPS < 70, and retreatment were confirmed in the multivariable model, while extent of resection (EOR) was not significant (HR = 1.36 [CI 95%, 0.59–3.12], *p* = 0.474) (Table 3).

**Figure 2:**
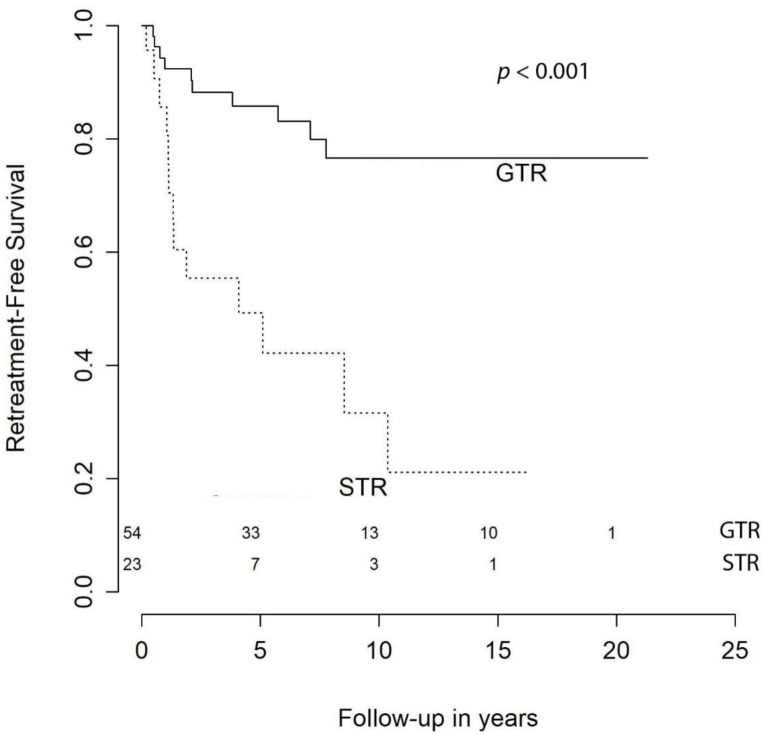
Retreatment-free survival by extent of resection.

In the present cohort, the retreatment-free survival at 1 year was 83.1%, at 2 years it was 74.0%, at 3 years it was 64.9%, at 5 years it was 51.9%, at 10 years it was 20.8%, and at 15 years it was 14.3%. Our univariate analysis demonstrates that STR (HR = 4.73 [CI 95%, 2.06–10.87], *p* < 0.001) and skull base location (HR = 2.67 [CI 95%, 1.17–6.06], *p* = 0.020) were negative predictors, even though only extent of resection (EOR) remained significant in the multivariable model (HR = 4.18 [CI 95%, 1.79–9.78], *p* < 0.001) (Figure 2; Table 3).

### 2.4 Predictors of Worsened Neurological Outcome

According to our univariate analysis, the only parameter that was associated with worsened neurological outcome at 6–12 months was STR (*p* = 0.044). It carried almost a six-fold greater risk of neurological outcome deterioration (OR = 5.8 [CI 95%, 1.22–27.63]). No other parameters were significant; therefore, no multivariable model was created.

## 3. Discussion

An intrinsic challenge for the painstaking investigation of clinical outcomes of atypical meningiomas is their low incidence across the population. As a result, studies often rely on aggregated cases over long periods of time to achieve sufficient power and follow-up duration for analysis. Studying these clinical entities has become even more problematic due to the shifting of WHO diagnostic criteria over time. When the 2000 WHO criteria were applied instead of the 1993 WHO criteria, the classification of around 30% of high-grade meningiomas changed, generally from a higher to a lower grade [22]. The 2007 WHO criteria introduced less of a paradigm shift in the classification of meningioma, but brain invasion remained ambiguously applied as a marker for atypical meningioma [23]. Finally, the 2016 WHO classification shed light on brain invasion which is now deemed to be a sufficient criterion for the diagnosis of WHO grade II meningioma [2]. Overall, more recent WHO classifications provide stronger correlations between grade and survival compared to the older ones. Hence, conclusions from older series should be interpreted with caution.

In our study, we present the surgical and neurological outcomes of a series of 77 consecutive intracranial atypical meningiomas. Furthermore, we analyzed the prognostic factors of OS and retreatment-free survival.

**Figure 3:**
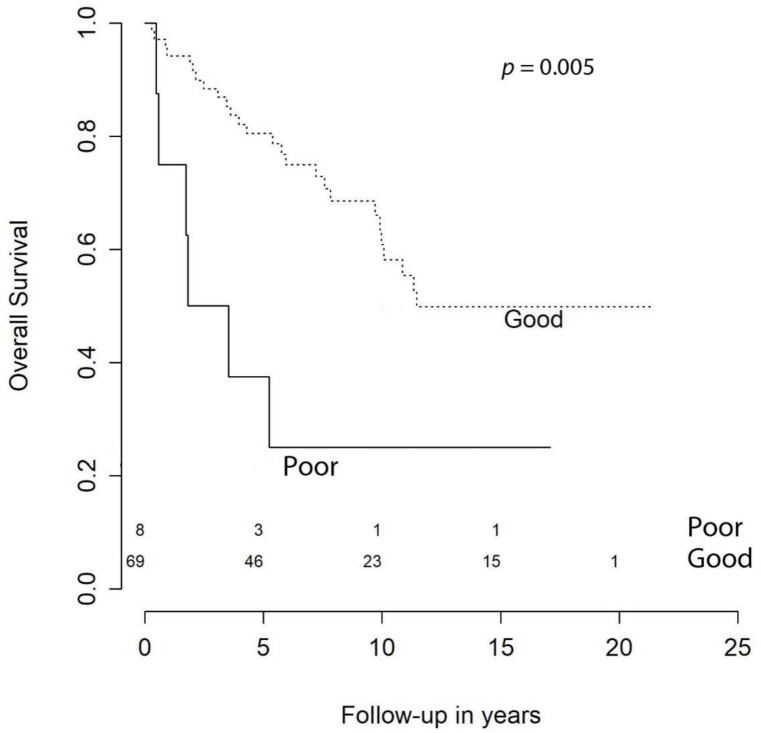
Overall survival by preoperative KPS.

As expected, our multivariable analysis identified age at surgery and preoperative KPS ≥ 70 as independent adverse prognostic factors for the OS (Figure 1 and Figure 3). With regard to age, we identified a 7% annual increase in risk of death of any cause and our results are in line with the current literature [10]. For instance, Streckert and colleagues [17] found that in their cohort, age was related negatively to OS and slightly positively correlated with recurrence. Contrarily, we failed to demonstrate a relationship between age and retreatment-free survival. This discrepancy may be explained by the fact that recurrence/progression and retreatment are not interchangeable. In fact, retreatment-free survival considers only those patients with progression/relapse that required a new intervention. Therefore, elderly patients may be excluded from candidacy for second procedures because of severe comorbidities. In our statistical analysis we could not avoid this confounding factor, since KPS at retreatment was not available. Hence, we could not include it in the multivariable model. In the literature, the correlation between patients’ age and recurrence remain controversial [19,24,25].

The role of KPS as a predictive factor in atypical meningiomas is poorly described in the literature. Goyal et al. [12] found that KPS < 80 was associated with neither reduced OS nor worse local tumor control. Similarly, in the study presented by Hammouche et al. [19] no association between poor KPS and increased recurrence ratio was detected. In our cohort, patients with KPS < 70 had a significant decreased OS compared to those with better KPS and, according to our multivariable analysis, this was independent from the age of the subject (Table 3).

In the present study, the definition of GTR included only Simpson grade I and II resections as described in the major publication on atypical meningiomas. Our GTR rate was 70.1% and was perfectly in line with the largest series in the literature that ranged between 48% and 87.3% [10,13,25,26,27].

Most authors suggest that EOR of atypical meningiomas is related to recurrence or progression [12,13,14,16,19,22,27,28]. Our findings confirm these authors’ results. Indeed, the patients in our cohort who underwent Simpson grade III–V resection had more than four times the risk of receiving retreatment (Figure 2; Table 3).

**Figure 4:**
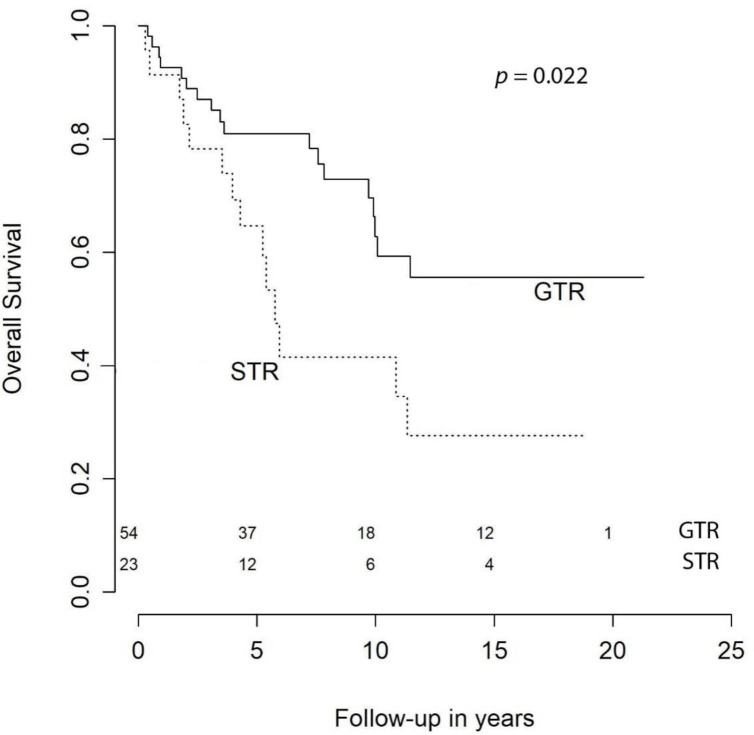
Overall survival by extent of resection.

More controversial is the impact of GTR on OS. The evidence available in the literature often shows no relationship [12,13] or just a trend towards shorter survival in partially resected atypical meningiomas [16,22,26]. In 2015, Aizer et al. [10] presented a large series of 575 atypical meningiomas and concluded that EOR is a powerful predictor of outcome for patients with atypical meningioma. According to the authors, their data highlighted the hazards associated with the presence of gross tumor bulk after surgery and suggested more extensive resections. Despite the magnitude of the publication, the study was limited in some respects. For instance, data on adjuvant RT were not available and therefore this potential confounding factor could not be ruled out in the multivariable model. Additionally, no central pathology review was undertaken, making the sample more heterogeneous. Our multivariable analysis failed to demonstrate the predictive power of EOR for OS (Figure 4). The significance detected in the univariate analysis is likely ascribable to age and KPS as confounding factors (Table 3). However, due to the impact of EOR on progression and recurrence, current guidelines recommend aiming at maximal safe resection also in atypical meningiomas [4]. Noteworthy, STR was the only statistically significant risk factor of worsened neurological outcome at 6–12 months (OR = 5.8 [CI 95%, 1.22–27.63], *p* = 0.044). Five out of 6 of the patients that experienced a decline in neurological status had a meningioma located at the skull base and all of them had neurological deficits at presentation. Three of them underwent a Simpson grade IV resection and one of them received only a biopsy of the lesion. One possible explanation is that STR in atypical meningiomas is often insufficient to improve the symptoms durably. In fact, the latest guidelines recommend the use of adjuvant RT whenever complete resection is not attainable [4]. None of these six patients received RT within 90 days of surgery. One of them was treated with conventional fractionated RT five months after surgery (Simpson grade IV). Two other patients received RT as retreatment some years later.

**Figure 5:**
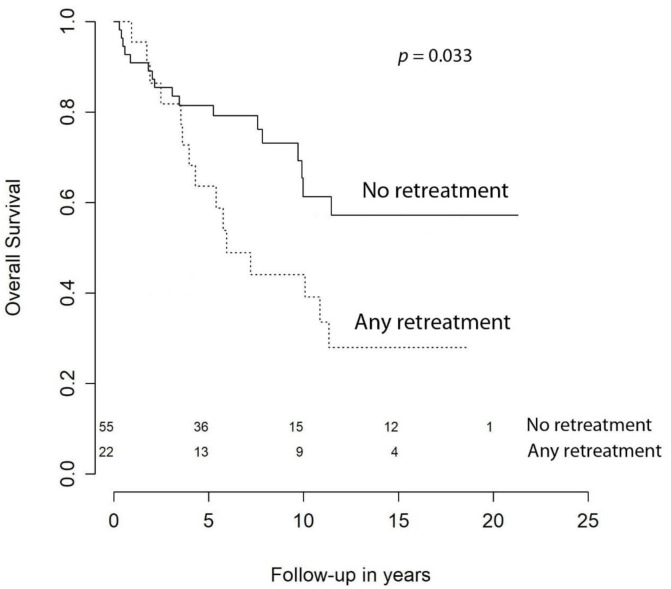
Overall survival by retreatment.

Almost all patients who underwent a second procedure for progression or recurrence of meningioma were treated with both surgery and adjuvant RT. This is nowadays sustained by solid evidence and the 2016 European Association of Neuro Oncology (EANO) guidelines state that in cases of progression, RT should be given with or without second surgery [4]. Interestingly, the comparison of median TTR from primary surgery to first retreatment and median TTR from first to second retreatment showed no significant difference (*p* = 0.836). Surprisingly, this is in contrast with what Lemée et al. [20] found in WHO grade I meningiomas. Indeed, they observed that TTR decreased significantly between surgeries in patients requiring repeated resections, indicating that surgical treatment of recurrences does not reset the clock but is indeed a “race against time”. Due to the paucity of patients requiring 4 or more surgical interventions in our cohort, we could not extend the comparison to further retreatments. However, even though the benefits of repeated excisions did not decrease with the number of surgeries in terms of TTR, patients who underwent retreatment had a significant reduction of OS. It can be postulated that those patients who underwent two or three surgeries were affected by more aggressive lesions compared to those who were treated just once. This adverse behavior may have an impact also on OS and the difference becomes evident from the fourth year of follow-up as suggested by the Kaplan–Meier curves (Figure 5). Unfortunately, data on biological markers, such as the Ki-67 index or MIB-1, were unavailable for a lot of patients and this impedes us further in our analysis of the molecular profile of our atypical meningiomas.

Regarding the predictive role of tumor location in atypical meningiomas, the literature is rather sparse and controversial. In the present study, we find no impact of tumor location dichotomized into skull base and non-skull base on OS and retreatment-free survival, even though a slight tendency towards more retreatments in SBM was identified (*p* = 0.09) (Table 3). On the contrary, Budohoski et al. [15] found parafalcine and parasagittal location positively associated with early recurrence within 24 months. In 2015, Klinger et al. [25] identified a higher risk of recurrence for atypical meningiomas found in the convexity location. However, these results are hardly comparable with ours since recurrences and retreatments are not synonyms. Given the strong correlation between EOR and retreatment-free survival and the loss of significance of tumor location in the multivariable model, it is likely that tumor location operates as a confounding factor and EOR is the only parameter with a solid predictive power.

## 4. Methods

### 4.1 Patient Cohort

We performed a review of a Norwegian population-based cohort of intracranial meningiomas treated surgically at the OUH, which is a tertiary referral center that captures all meningioma patients within an area with approximately 3 million inhabitants (56% of the Norwegian population). A total of 77 consecutive patients who underwent craniotomies for intracranial atypical meningioma (WHO grade II) between 1990–2010 were investigated. Clinical information was retrospectively reviewed using patients’ medical and surgical records from 1990 to 2002, whereas patients’ data from 2003 to 2010 were prospectively collected. KPS [29] was assessed using clinical records of preoperative visits. The age cutoff used to dichotomize the cohort into elderly and younger patients was set at 65 years based on the report from Ostrom et al. [1], which identified a dramatic increase in meningioma incidence in those aged over 65. The following variables were registered: gender, age, presence of neurological deficits, tumor location, histology, and surgical outcomes.

### 4.2 Tumor Characteristics

The preoperative post-contrast imaging studies were reviewed to confirm location, contrast enhancement, calcification, and size of the tumors. Each surgical case was approached attempting GTR. The WHO grading system was used to classify the histology of meningiomas. The WHO criteria changed during the study period. From 1990 to 2001, the tumors were classified as benign, atypical or anaplastic. In 2001, a WHO-grading system for meningioma, which divides the tumors into grade I, II, and III, was implemented. The grading system was changed again in 2016 [2], and brain invasion is now deemed to be a sufficient criterion for the diagnosis of WHO grade II. Previously, these tumors were all classified as WHO grade I. For this study, we reclassified all lesions according to the 2016 WHO classifications. The definition of SBM was based on Al-Mefty et al. [21] and thus every intracranial meningioma located elsewhere was considered a non-skull base meningioma NSBM [8]. The bone invasion was assessed only radiologically using preoperative CT and/or MRI imaging. Histological analyses were not routinely performed to evaluate the diseased bone.

### 4.3 Outcome

The surgical management aimed at achieving GTR whenever possible. The EOR was assessed using the Simpson grade scale. GTR was defined as Simpson grade I or II in accordance with major publications on atypical meningiomas, while Simpson grade III, IV, and V were classified as STR. CT scans and MRIs were also reviewed to confirm the degree of tumor removal. Neurological status at 6–12 months after surgery was dichotomized into improved-stable or worsened compared to preoperative status. Early post-operative complications were defined as postoperative on-site hematoma or surgical site infection requiring a second surgery, regardless of timeline, corresponding at least to a grade IIb complication, according to the Landriel Ibanez classification [30]. Only recurring tumors with radio-clinical correlations, occurring at the site of the previous surgery and requiring surgical retreatment were included in the retreatment cohort. Radiological recurrences without clinical correlates, thus not requiring any adjuvant treatment, as well as lesions occurring at locations other than the primary site of the tumor, were instead considered non-retreated patients. To avoid subjectivity in differentiating postsurgical tumor remains from scars located near the resection sites, the retreatment-free survival was defined as the time between the first surgery and the first subsequent retreatment (either RT or a new surgical procedure). Adjuvant RT was defined as RT within 3 months of surgery, not in the context of recurrence. Vital status (alive or dead) and time of death was obtained from the Norwegian Population Registry (Folkeregisteret) on 21 January 2011. OS was calculated from the time of primary surgery to the time of death or censoring. Retreatment-free survival was calculated from the time of primary surgery to the time of retreatment, time of death, or censoring.

### 4.4 Ethics

The study is regulated by the Personal Data Act/Personal Health Data Filing System Act and approved by the Data Protection Official at OUH (2017/5204). Informed consent is not required by the Personal Data Act/Personal Health Data Filing System Act.

### 4.5 Statistics

Descriptive analysis was carried out using median and IQR for the quantitative variables and percentage values for the qualitative ones. Normality distribution was assessed by the Shapiro–Wilk test. The association between qualitative variables was investigated using Pearson’s chi-squared or Fisher’s exact test while the non-parametric Wilcoxon rank-sum test was used for quantitative variables. The comparison of the median values of the TTR variable in patients who required repeated surgeries was carried out using the Wilcoxon test for independent samples. Survival analysis was performed by applying the Kaplan–Meier estimator and log-rank test for equality of survivor functions. The association with clinical features was analyzed with the Cox model of proportional hazards (hazard ratio (HR) and 95% CI), and the applicability assumption was evaluated by the Schoenfeld test. Statistical significance was set at < 0.05. All analyses and graphical drawing were performed using R v3.6.3 (https://www.r-project.org).

## 5. Conclusions

In our study, GTR did significantly prolong retreatment-free survival but had no significant impact on OS. GTR was also associated with improved/stable neurological outcome at 6–12 months. Age at surgery, preoperative KPS, and retreatment were all strong prognostic factors of OS. Furthermore, we observed that TTR did not decrease significantly in patients requiring repeated surgical excision. This suggests that surgical retreatment may be of help in certain patients, though it is unable to restore the baseline situation as suggested by the reduction of OS in retreated patients.

## 6. Strengths and Limitations of the Study

The strength of this study is its long and complete follow-up (median = 5.75 [IQR: 8.14] years). The study includes all craniotomies performed for histologically confirmed WHO grade II meningiomas. A central pathology review was undertaken to reclassify all lesions according to the latest WHO criteria. The pre- and postoperative postcontrast imaging studies were reviewed to confirm tumor location and EOR. With respect to data quality, we only used end-points that are easily verifiable (i.e., 30-day mortality, reoperation for hematomas, and reoperations for infections).

However, this study is not free from limitations. First of all, due to the retrospective nature of our analysis, there are limitations in terms of data collection inherent in such studies, despite data from 2003 being collected prospectively. For instance, data about adjuvant RT were not available for all patients. Thus, data on dosage and number of fractions were not included. Secondly, the single-center design of the study greatly limits the generalizability of our results. Moreover, the cause of death was not available for every patient and hence we cannot calculate the disease-specific survival and the OS includes mortality due to any cause. Neither the Ki-67 index nor the MIB-1 staining index was available for the majority of the tumors and this parameter was therefore excluded from the study. Only surgical mortality, the rate of postoperative hematoma, and the rate of deep postoperative infection were used in this study as indicators for quality of surgery.

## Figures and Tables

**Table 1:** Overall characteristics of patients involved in the study.

	*n*	%
	77	100
Sex		
Male	31	40.3%
Female	46	59.7%
Age at Primary Surgery		
Median [IQR]	62.21 [22.87] years	
Preoperative KPS		
100	2	2.6%
90	20	26.0%
80	28	36.4%
70	19	24.7%
<70	8	10.4%
Symptoms at Presentation		
Neurological deficits	51	66.2%
Raised ICP	36	46.8%
Seizures	21	27.3%
Asymptomatic	1	1.3%
Location		
Convexity	22	28.6%
Falx	12	15.6%
Parasagittal	19	24.7%
CP angle	4	5.2%
Lateral sphenoid wing	5	6.5%
Medial sphenoid wing	3	3.9%
Olfactory groove	2	2.6%
Petroclival	1	1.3%
Tentorium-intra	3	3.9%
Tentorium-supra	2	2.6%
Tuberculum sellae/suprasellar	2	2.6%
Intraventricular	2	2.6%
Bony Invasion		
Yes	17	22.1%
No	60	77.9%
Multiple Meningiomas		
Yes	5	6.5%
No	72	93.5%

Note: CP—cerebellopontine; ICP—intracranial pressure; IQR—Interquartile range; KPS—Karnofsky performance status. * Quantitative variables are expressed using median and IQR, while the categorical variables are expressed using absolute numbers and percentages.

**Table 2:** Therapeutic management of atypical meningiomas.

	*n*	%
	77	100
Simpson Grade		
Grade I	35	45.5%
Grade II	19	24.7%
Grade III	6	7.8%
Grade IV	16	20.8%
Grade V	1	1.3%
30-Day Mortality		
	0	0.0%
Early Postoperative Complications		
Hematomas	2	2.6%
Infections	4	5.2%
Neurological Outcome at 6–12 Months		
Improved/stable	39	50.6%
Worsened	9	11.7%
No data	29	37.7%
Retreatment		
Any retreatment	22	28.6%
Radiotherapy only	1	1.3%
Surgery only	1	1.3%
Surgery and radiotherapy	20	26.0%
Early Postoperative Complications after Retreatment		
Hematomas	0	0.0%
Infections	1	4.5%

* Quantitative variables are expressed using median and IQR, while the categorical variables are expressed using absolute numbers and percentages.

**Table 3:** Univariable and multivariable Cox analyses for overall survival and retreatment-free survival.

	Cox Model for Overall Survival			
	Univariable Analysis		Multivariable Analysis	
	HR (95% CI)	*p*-Value	HR (95% CI)	*p*-Value
**Age at Primary Surgery**	1.07 (1.03–1.10)	<0.001	1.08 (1.04–1.12)	**<0.001**
**Preoperative KPS** *Poor vs. Good*	3.70 (1.51–9.09)	0.005	4.00 (1.49–11.11)	**0.006**
**Meningioma Requiring Retreatment** *Any retreatment vs. No Retreatment*	2.13 (1.06–4.28)	0.033	3.30 (1.49–7.32)	**0.033**
**Extent of Resection** *STR vs. GTR*	2.28 (1.12–4.60)	0.022	1.36 (0.59–3.12)	0.474
**Location** *Skull base vs. non skull base*	0.90 (0.42–1.95)	0.790	-	-
**Bony Invasion** *Yes vs. No*	0.82 (0.35–1.90)	0.635	-	-
**Sex** *Female vs. Male*	0.67 (0.34–1.35)	0.262	-	-
**Multiple Meningiomas** *Single vs. multiple*	0.90 (0.21–3.82)	0.890	-	-
	**Cox Model for Retreatment-Free Survival**			
	**Univariable Analysis**		**Multivariable Analysis**	
	**HR (95% CI)**	***p*-Value**	**HR (95% CI)**	***p*-Value**
**Age at Primary Surgery**	0.98 (0.95–1.01)	0.270	-	-
**Preoperative KPS** *Poor vs. Good*	0.75 (0.18–3.23)	0.704	-	-
**Extent of Resection** *STR vs. GTR*	4.73 (2.06–10.87)	<0.001	4.18 (1.79–9.78)	**<0.001**
**Location** *Skull base vs. non skull base*	2.67 (1.17–6.06)	0.020	2.09 (0.90–4.84)	0.09
**Bony Invasion** *Yes vs. No*	0.52 (0.21–1.27)	0.152	-	-
**Multiple Meningiomas** *Single vs. multiple*	1.29 (0.17–9.60)	0.804	-	-

Note: CI—confidence interval; GTR—gross total resection; HR—hazard ratio; KPS—Karnofsky performance status; SG—Simpson grade; STR—subtotal resection.

## Data Availability

The datasets generated and/or analyzed during the current study are available from the corresponding author on reasonable request.
